# Cutaneous Blastomycosis Mimicking Breast Malignancy

**DOI:** 10.7759/cureus.17276

**Published:** 2021-08-18

**Authors:** Rose Chisenga, Tulika Chatterjee, Zita Erbowor-Becksen, Pranitha Kovuri, Abuzar A Asif

**Affiliations:** 1 Internal Medicine, University of Illinois College of Medicine, Peoria, USA

**Keywords:** cutaneous blastomycosis, breast malignancy, blastomycosis, infection, mimic

## Abstract

Blastomycosis is a fungal infection caused by *Blastomyces dermatitidis*. While blastomycosis can cause systemic infection affecting multiple organs, localized blastomycosis of the breast is uncommon. Here, we report the case of a 50-year-old female with a localized left breast growth which started as a nodule and later ulcerated extensively. Although her clinical picture raised concerns for breast malignancy, workup revealed cutaneous blastomycosis with superimposed methicillin-susceptible *Staphylococcus aureus* and *Klebsiella oxytoca* infection. Interestingly, there was no evidence of pulmonary disease on CT chest imaging. She was treated with Amphotericin B for seven days and discharged on oral Itraconazole for nine months. Additionally, she received amoxicillin-clavulanate for her bacterial superinfection. On the six-month follow-up, the patient showed significant improvement. Blastomycosis can mimic several diseases including malignancy, pyoderma gangrenosum, and mycobacterial and bacterial infections leading to delayed diagnosis and treatment.

## Introduction

Blastomycosis, caused by *Blastomyces dermatitidis*, is a common endemic mycosis that occurs predominantly in North America including the northcentral, midwestern, and southern states of the United States [1]. The annual incidence of blastomycosis in the United States has been estimated at 1-40 cases per 100,000; however, as only a few states report it, the true annual incidence remains unknown [2]. The primary infection mainly involves the lungs. Most infections are either asymptomatic or accompanied by mild respiratory symptoms that are usually attributed to nonfungal respiratory conditions [1]. The primary pulmonary infection can subsequently spread hematogenously to the skin, bone, genitourinary system, and central nervous system (CNS). Cutaneous cases of blastomycosis usually originate from a pulmonary site regardless of whether a patient presents with a clinically apparent lung infection; however, in rare cases, direct cutaneous inoculation with *B. dermatitidis* has been reported [3]. Blastomycosis is a great mimicker of various medical conditions and pathologies, including neoplasia, mycobacterial infections, immunological skin disorders, and other mycoses, which can make diagnosis even more challenging [4].

## Case presentation

**Figure 1:**
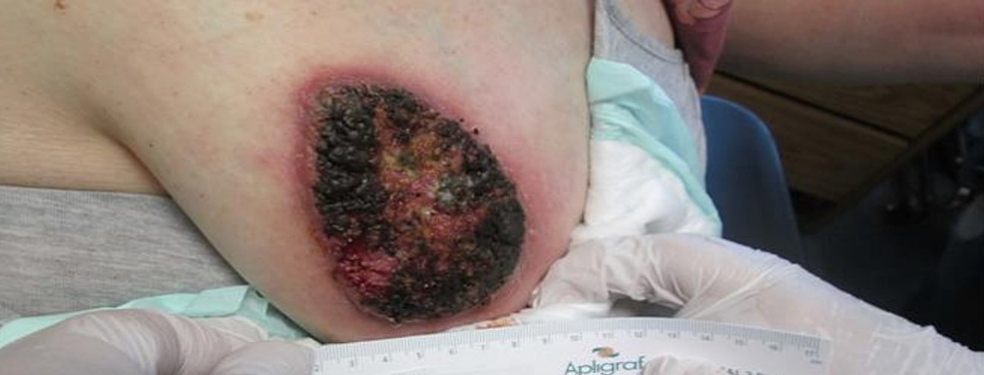
Left breast lesion prior to hospitalization.

**Figure 2:**
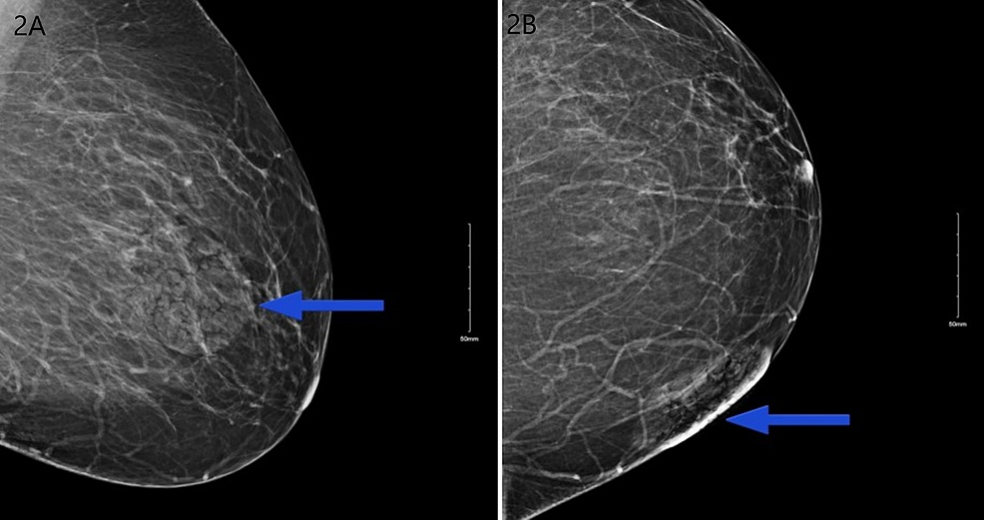
Mammogram showing breast lesion confined to the skin (lesion is marked with a blue arrow).

**Figure 3:**
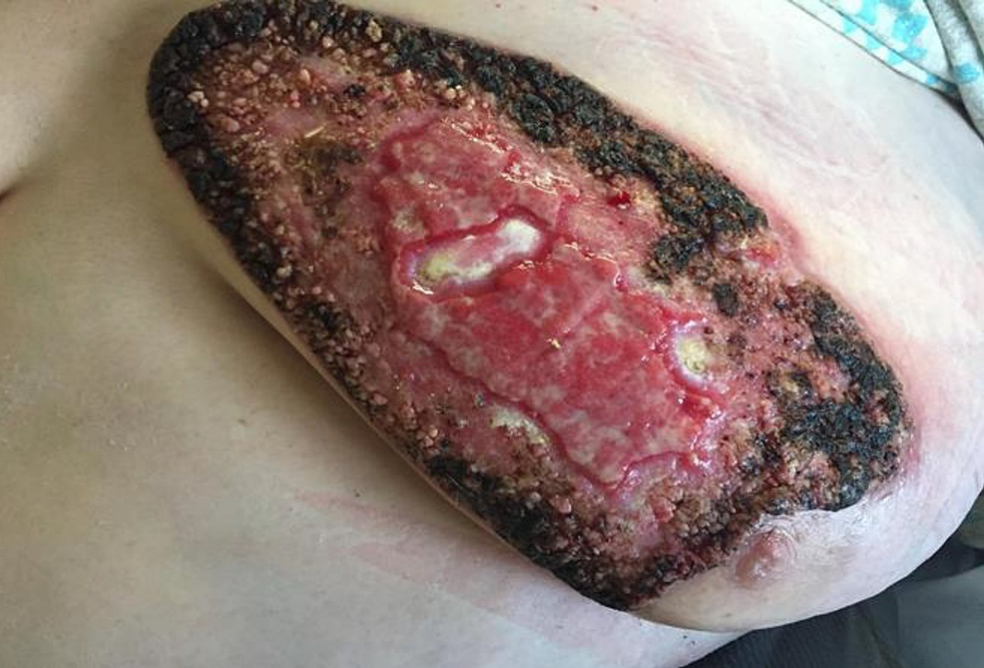
Left breast lesion on the day of hospitalization.

A 50-year-old female with a past medical history of type 2 diabetes mellitus, hypertension, morbid obesity (body mass index 51.4 kg/m^2^), and hyperlipidemia presented with a left breast growth that she had first noticed a year ago. It started as a nodule which ulcerated after three weeks (Figure 1). The area became progressively tender, erythematous, and eventually affected more than half of her breast. She received multiple outpatient trials of topical and oral antibiotics without improvement. The patient had obtained a mammogram as an outpatient which reported the breast abnormality as a “superficial skin lesion without any breast parenchymal abnormality” (Figures 2A, 2B). She also reported a foul-smelling discharge and occasional left breast itchiness lasting two to three weeks prior to presentation. The patient denied any history of fever, night sweats, weight loss, trauma to the breast, or any other skin abnormalities. She had no history of cough, hemoptysis, or other pulmonary symptoms. Her family history was remarkable for breast carcinoma in her grandmother diagnosed after age 60. She had predominantly lived in the midwestern part of the United States but denied recent travel, sick contacts, hiking to wooded areas, or insect bites. She had healthy cats and dogs for pets with no reported animal bites. On physical examination, the patient’s vital signs were within normal limits and she had a verrucous-appearing growth on her left breast measuring approximately 20 cm × 10 cm with raised irregular borders and a foul-smelling discharge. There was a small rim of peri-lesion erythema, and the lesion eroded the areola with no nipple retraction (Figure 3). Palpation revealed no breast lumps or lymphadenopathy.

**Figure 4:**
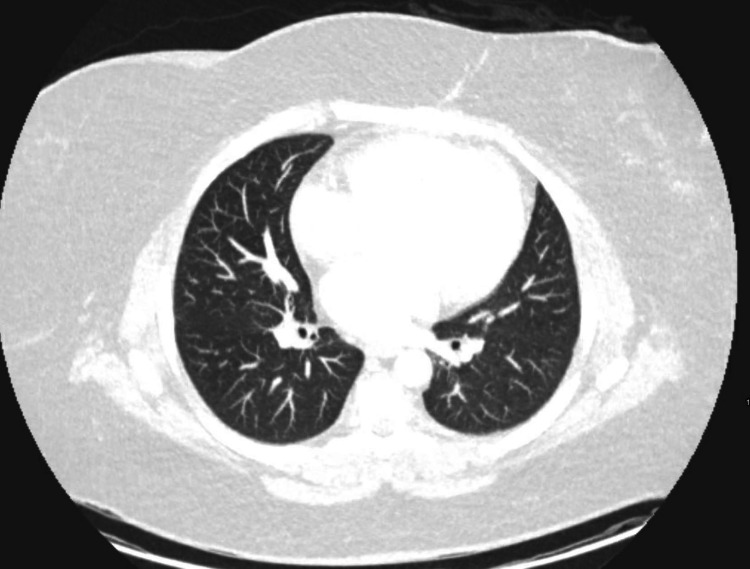
Transaxial chest CT with no evidence of pulmonary fungal disease. CT: computed tomography

Initial laboratory tests revealed no significant findings except for an elevated C-reactive protein and a positive serum *Blastomyces* quantitative antigen test (Table 1). Blood fungal cultures were negative. Biopsy and culture of the breast tissue confirmed blastomycosis with superimposed bacterial infection (methicillin-susceptible *Staphylococcus aureus *and *Klebsiella oxytoca*). To further rule out systemic blastomycosis, chest and brain imaging were scheduled. CT chest showed no evidence of pulmonary fungal disease (Figure 4). MRI brain was not obtained because of the patient’s severe claustrophobia. It is important to note that the patient had no neurological symptoms.

**Table 1:** Laboratory findings. Ab: antibody; Ag: antigen

Laboratory test	Value	Reference range
White blood cell count	7.94 × 10^3^/µL	4.00–12.00 × 10^3^/µL
Red blood cell count	5.11 × 10^6^/µcL	3.80–5.30 × 10^6^/µL
Hematocrit	42.8%	36.0–47.0%
Hemoglobin	13.9 g/dL	12.0–15.8 g/dL
Mean corpuscular volume	83.8 fL	82.0–96.0 fL
Platelet count	225 × 10^3^/µL	140–440 × 10^3^/µL
Neutrophils	65.1%	47.0–73.0%
Lymphocytes	26.6%	18.0–42.0%
Monocytes	6.7%	4.0–12.0%
Eosinophils	1.1%	0.0–5.0%
Basophils	0.5%	0.0–1.0%
Absolute neutrophils	5.17 × 10^3^/µL	1.60–7.70 × 10^3^/µL
Absolute lymphocytes	2.11 × 10^3^/µL	1.30–3.20 × 10^3^/µL
Sodium	134 mmol/L	136–145 mmol/L
Potassium	3.7 mmol/L	3.5–5.1 mmol/L
Creatinine	0.73	0.60–1.00 mg/dL
C-reactive protein	4.51 mg/dL	<0.50 mg/dL
Histoplasma capsule H Ab	Negative	
Histoplasma capsule M Ab	Negative	
Coccidioides immitis Ab	Negative	
Blastomyces dermatitidis Ab	Negative	
Aspergillus fumigatus Ab	Negative	
Beta-D-glucan (1,3) (Fungitell)	Negative	
Serum Blastomyces quantitative Ag	Positive	
HIV 1, 2 Ag Ab screen	Negative	

The patient was treated with Amphotericin B for seven days and discharged on oral Itraconazole. Additionally, she received amoxicillin-clavulanate for 10 days for superimposed bacterial infections.

On the six-month follow-up, the patient showed significant improvement with near resolution of the skin abnormality while on antifungal therapy, and the course of Itraconazole was reduced to a total of nine months.

## Discussion

Blastomycosis mimics several diseases and can be easily misdiagnosed as neoplasia, pyogenic gangrenosum, nocardiosis, mycobacterial infection, immunologic skin disorders, and other mycoses [4]. In the spectrum of the presentation of blastomycosis, breast involvement is uncommon, as noted in the literature [5-9].

The most common clinical presentation of blastomycosis is pneumonia though pulmonary infiltrates may be found on routine chest radiographs of patients with blastomycosis despite a lack of respiratory complaints [6,8]. Cutaneous involvement is the next most common clinical presentation [6]. Cutaneous lesions of blastomycosis may be verrucous or ulcerative in nature [6,9]. Verrucous lesions are raised and crusted with irregular sharp borders and underlying subcutaneous abscesses [10]. Skin ulcerations develop due to spontaneous rupture of the subcutaneous abscesses [6]. Our patient initially reported a “pimple-like” skin abnormality, which was likely a subcutaneous blastomycosis abscess which later ruptured and ulcerated. Edges of blastomycosis cutaneous ulcers are sharp and raised along with central exudative regions, as was evident in our patient’s breast lesion.

Most cases of cutaneous blastomycosis occur following the lymphohematogenous spread of pulmonary blastomycosis. With time, the patient develops an immune response which leads to pyogranulomatous changes at the site of primary or secondary inoculation which is followed by the formation of noncaseating granulomas [10]. About half of secondary cutaneous cases of blastomycosis have no evidence of pulmonary infection at the time of diagnosis and have normal chest radiographs. Most patients with cutaneous blastomycosis who have no evidence of pulmonary infection are presumed to have had a subclinical pulmonary infection or an asymptomatic phase that had already resolved. Because cutaneous blastomycosis is usually associated with negative chest radiography, it is difficult to distinguish between primary and secondary cutaneous blastomycosis with a resolved or subclinical pulmonary phase of the disease [11]. In this case, it was difficult to determine whether it was a case of primary or secondary cutaneous blastomycosis.

As noted in our case, diagnosis of cutaneous blastomycosis can be challenging. Urine and serum blastomycosis antigen tests have good sensitivity but are not as specific due to cross-reactivity with other systemic mycoses, particularly histoplasmosis. Microscopic visualization and tissue culture are more specific; however, tissue culture can take up to five weeks causing a delay in diagnosis. Microscopic visualization is timely but less sensitive than culture, requiring a good sample size and local expertise. Although polymerase chain reaction and DNA sequencing tests such as Karius are newer noninvasive diagnostic modalities that can be used, they need further large-scale investigation to establish their real-world clinical impact. It is important to utilize multiple testing modalities available for accurate and timely diagnosis of cutaneous blastomycosis [12-14].

## Conclusions

This case elaborates how cutaneous blastomycosis can mimic different pathologies including malignancy depending on its location. Skin abnormalities have been reported in 40-80% of blastomycosis cases. The unusual location of the lesion on the breast made the diagnosis challenging in our case. This case emphasizes the importance of combining antigen test results, prompt tissue biopsy, and culture whenever fungal mycoses are suspected. Treatment with Amphotericin B followed by Itraconazole has been shown to be highly effective. Even though most cases respond well to oral Itraconazole for 6-12 months, disseminated blastomycosis cases including those involving CNS require intravenous Amphotericin B before starting oral Itraconazole.
